# Effects of home‐cage elevation on behavioral tests in mice

**DOI:** 10.1002/brb3.3269

**Published:** 2023-12-08

**Authors:** Hiroshi Ueno, Yu Takahashi, Shinji Murakami, Kenta Wani, Yosuke Matsumoto, Motoi Okamoto, Takeshi Ishihara

**Affiliations:** ^1^ Department of Medical Technology Kawasaki University of Medical Welfare Okayama Japan; ^2^ Department of Psychiatry Kawasaki Medical School Kurashiki Japan; ^3^ Department of Neuropsychiatry, Graduate School of Medicine Dentistry and Pharmaceutical Sciences Okayama University Okayama Japan; ^4^ Department of Medical Technology, Graduate School of Health Sciences Okayama University Okayama Japan

**Keywords:** behavior, behavioral test, mouse, home‐cage, anxiety

## Abstract

**Background:**

Research reproducibility is a common problem in preclinical behavioral science. Mice are an important animal model for studying human behavioral disorders. Experimenters, processing methods, and rearing environments are the main causes of data variability in behavioral neuroscience. It is likely that mice adapt their behavior according to the environment outside the breeding cage. We speculated that mice housed on elevated shelves and mice housed on low shelves might have differently altered anxiety‐like behavior toward heights.

**Purpose:**

The purpose of this study was to investigate potential behavioral changes in mice raised at different heights for 3 weeks. Changes in behavior were examined using various experimental tests.

**Results:**

Mice housed on elevated shelves showed reduced anxiety‐like behavior in a light/dark traffic test compared with mice housed on low shelves. There were no significant differences between the two groups in terms of activity, exploratory behavior, muscle strength, or depression‐like behavior.

**Conclusions:**

Our results indicate that different cage heights and corresponding light exposure may alter the anxiety‐like behavior of mice in response to brightness. Researchers need to carefully control the cage height and light intensity experienced by the mice to produce reproducible test results.

## Graphical abstract

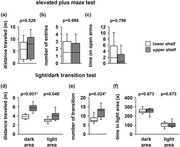

## 1 INTRODUCTION

Mice (*Mus musculus*) are the most commonly used animal species in biomedical research. Laboratory mice play a central role in the study of animal models of human behavioral disorders (Crawley, 2007). Several laboratories worldwide have used genetically defined identical mouse strains and mutant mice to address complex behavioral problems. However, in recent years, there has been a growing interest in the reproducibility of behavioral phenotypes in mice (Kafkafi et al., 2018). For the successful transfer of the obtained results to human experiments, it is necessary to standardize and properly report the appropriate handling, handling methods, and breeding methods for laboratory mice.

Study reproducibility (Goodman et al., 2016) is a growing concern in preclinical behavioral science (Loken & Gelman, 2017; Steckler et al., 2015; Voikar, 2020). Similar to other areas of biomedical research, preclinical studies must be reproducible, especially when dealing with laboratory animal behavior, which is highly sensitive to environmental factors (Olsson & Dahlborn, 2002; Sousa et al., 2006). Standardized environmental conditions should be used to reduce variability between animals in the same experimental group and between studies, facilitate detection of treatment effects, and increase reproducibility of results across laboratories (Van Zutphen et al., 2001). The experimenter (Bohlen et al., 2014) and processing methods (Gouveia & Hurst, 2017) have been shown to be major contributors to data variability in behavioral neuroscience.

In animal research, the cage environment and housing procedures are designed to ensure animal welfare and health. Mice are typically housed in transparent “shoebox” cages containing bedding, food, and water. Laboratory housing conditions are primarily based on economic (minimum use of space, equipment, and labor), ergonomic (easy handling, animal visibility), and hygiene (easy disinfection) considerations as well as standardization (Baumans & Van Loo, 2013; Olsson & Dahlborn, 2002), which can affect the experimental results and reproducibility and lead to confounding effects (Festing, 2020). In their home‐cages, mice perform natural, spontaneous, and often complex behaviors such as playing, caring, socializing, and nest‐building (Jirkof, 2014). It has been reported that experimental mice change their behavior based on visual, olfactory, and auditory information. The phenotypes of mice can change depending on their home‐cag environment. We have previously shown that the home‐cag environment also influences data variability in behavioral neuroscience (Ueno et al., 2020). However, many studies have poorly described the properties of the home‐cages used.

Mice recognize cagemates that exhibit abnormal behavior using visual and olfactory information (Langford et al., 2006; Smith et al., 2016; Ueno et al., 2018) and show interest in them (Yang et al., 2016). They are social animals and have a strong tendency to follow individuals of the same species (Hedrich & Bullock, 2004). It has also been reported that mice can visually distinguish between photographs (Watanabe, 2013) and can perceive virtual reality spaces (Drew, 2019). In sum, these reports indicate that mice recognize the environment outside their home‐cage which influences their behavioral phenotypes.

Behavior, which represents the ultimate output of the nervous system of all organisms, results from the interaction between genotype and environment. Therefore, the measurement of behavioral outcomes is essential for characterizing animal models of neurodegenerative and neuropsychiatric disorders. In general, depression‐like behavior, aggression, activity, and anxiety‐like behavior in mice are measured through a series of behavioral experiments to analyze the effects of drugs and other stimuli (Wahlste, 2010). Behavioral test batteries have been used to assess a variety of behavioral traits, including motor activity, sensory and motor function, anxiety‐like behavior, learning, and memory in inbred as well as mutant mice strains (Crawley, 2008; Crawley & Paylor, 1997). Over the last 25 years, behavioral phenotyping of transgenic mice has become a commonly used approach in behavioral neuroscience and genetics (Voikar, 2020).

C57BL/6, which were used in the present study, and DBA/2J mice are the oldest and most commonly used inbred strains in behavioral genetics (Bryant et al., 2008), as they are considered to exhibit a moderate phenotype in many behavioral domains (Crawley et al., 1997).

The purpose of the current study was to evaluate the effect of home‐cage elevation on the behavioral phenotypes of mice. For this purpose, we housed mature mice in rearing cages and created groups that were either housed in elevated or in low positions. After 3 weeks, the mice were adapted to a behavioral test battery and examined for behavioral abnormalities. We hypothesized that the elevated‐cage group would show a reduction in anxiety‐like behavior toward high altitudes.

## 2 MATERIALS AND METHODS

### 2.1 Ethics statements

All animal experiments were performed in accordance with the ARRIVE guidelines (http://www.nc3rs.org.uk/arrive‐guidelines) and the U.S. National Institutes of Health (NIH) Guide for the Care and Use of Laboratory Animals (NIH Publication No. 80‐23, revised in 1996) and were approved by the Committee for Animal Experiments at the Kawasaki Medical School Advanced Research Centre. No statistical methods were used to predetermine the required sample size. All efforts were made to minimize the number of animals used and their suffering.

### 2.2 Animals

Eight‐week‐old C57BL/6N male mice were purchased from CLEA Japan and housed in cages (C57BL/6N mice: five animals per cage) with food and water provided ad libitum under a 12 h light/dark cycle at 23–26°C. All mice were maintained on a 12 h (8:00 a.m.–8:00 p.m.) light cycle and a 12 h (8:00 p.m.–8:00 a.m.) dark cycle. Considering that behavioral variability is partially sex‐dependent, and that comparing the behavior of males versus females was not the purpose of this experiment, only male mice were included in the study.

### 2.3 Rearing on shelves of different heights

**FIGURE 1:**
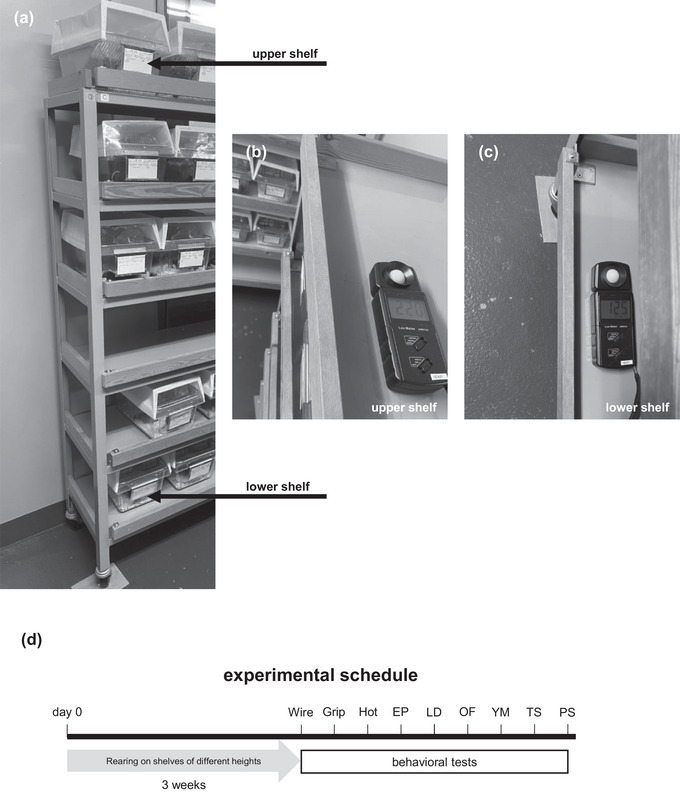
Home‐cage shelf heights and illuminances. (a) Upper shelf group: The home‐cage was placed at a height of 165 cm. Lower shelf group: The home‐cage was placed at a height of 20 cm. The illuminance for the upper shelf group was 220 lx during the light period (b). The illuminance for the lower shelf group was 125 lx during the light period (c). (d) Experimental time schedules. Over the course of 3 weeks, the two groups experienced shelves of different heights. Afterward, we performed the behavioral test battery. Mice were subjected to one behavioral test per day. EP, elevated plus maze test; Grip, grip strength test; Hot, hot plate test; LD, light/dark transition test; OF, open field test; PFST, Porsolt forced swim test; TS, tail‐suspension test; Wire, wire hang test; YM, *Y*‐maze test.

The mice were randomly divided (http://www.randomizer.org) into two groups: the upper and lower shelf groups (Figure 1a). Over the course of 3 weeks, the two groups experienced shelves of different heights. Then, the mice were subjected to behavioral tests. (1) For the upper shelf group, the home‐cage was placed at a height of 165 cm, and the illuminance at this height was 220 lx during the light period (Figure 1b). (2) For the lower shelf group, the home‐cage was placed at a height of 20 cm, and the illuminance at this height was 125 lx during the light period (Figure 1c).

### 2.4 Behavioral tests

All behavioral tests were conducted in behavioral testing rooms between 09:00 and 16:00 h during the light phase of the light/dark cycle (Ueno et al., 2022). Each test was separated from the next by at least 1 day. One behavioral test was performed consecutively in 1 day. We tested the mice in random order. After the tests, the equipment was cleaned with 70% ethanol and superhypochlorous water to prevent artifacts caused by lingering olfactory cues. Behavioral tests were performed on naïve mice in accordance with the test order described below.

### 2.5 Wire hang test

In the wire hang test, the mice were placed on a wire mesh that was then inverted and waved gently so that they gripped the wire. A wire hang test apparatus (O'Hara & Co.) was used, and the latency to fall was recorded.

### 2.6 Grip strength test

Neuromuscular strength was examined using the grip strength test. Forelimb strength was measured using a grip strength meter. The mice were lifted and held by the tail so that their forepaws could grasp a wire grid. Subsequently, they were gently pulled back until they released the grid. The peak force applied by the forelimbs was recorded in Newtons (cN).

### 2.7 Hot plate test

The hot plate test was used to evaluate nociception. The mice were placed on a plate heated to 55.0 ± 0.3°C, and the latency to the first paw response was recorded. Paw responses included foot shakes or paw licks. A latency period of 30 s was defined as complete analgesia and used as the cut‐off time to prevent tissue injury.

### 2.8 Elevated plus maze test

Anxiety‐like behavior was examined using the elevated plus maze. The apparatus consisted of two open arms (8 × 25 cm) and two closed arms of the same size, with 30‐cm‐high transparent walls. The arms were constructed of white plastic plates and elevated to a height of 40 cm above the floor. Arms of the same type were located the opposite to each other. Each mouse was placed in the central square of the maze, facing a closed arm, and allowed to move freely among the four arms for 6 min. The mice were video‐recorded, and the number of arm entries, distance traveled (m), and time spent in the open arms were recorded using video tracking software (ANY‐MAZE, Stoelting Co.).

### 2.9 Light/dark transition test

The light/dark transition test was performed as previously described (Takao & Miyakawa, 2006). The apparatus consisted of an acrylic cage (22 × 44 × 40 cm^3^) divided into two sections of equal size by a partition with a door. One chamber had white acrylic walls and was brightly illuminated (200 lx) by lights above the ceiling of the chamber, whereas the other chamber had black acrylic walls and was dark (50 lx). Both chambers had white plastic floors. The mice were placed in the dark chamber and allowed to move freely between the two chambers for 6 min with the door open. The distance traveled (m), total number of transitions, and time spent in the light chamber (s) were recorded using the ANY‐MAZE software.

### 2.10 Open field test

Exploratory behavior, anxiety‐like behavior, and general locomotor activity were examined using an open field test. Each mouse was placed in the center of an apparatus consisting of a square area surrounded by walls (45 × 45 × 40 cm^3^). The distance traveled (m), number of entries into the central area, and time spent in the central area (s) were recorded. The central area was defined as the middle 20 × 20 cm^2^ portion of the field. The test chamber was illuminated at 100 lx. Data were collected over a 30 min period using the ANY‐MAZE software.

### 2.11 *Y*‐maze test

Spatial working memory was measured using a *Y*‐maze apparatus (arm length: 40 cm; arm bottom width: 3 cm; upper arm width: 10 cm; wall height: 12 cm). The mice were placed in the center of the *Y*‐maze for 6 min. Visual cues were placed around the maze in the testing room and they remained constant throughout the testing sessions. The mice were tested with no previous exposure or habituation to the maze. The total distance traveled (m), number of entries, and number of alternations were recorded using ANY‐MAZE software.

### 2.12 Tail‐suspension test

Depressive‐like behavior was examined using the tail‐suspension test (Ueno et al., 2022). Each mouse was suspended by its tail in a white plastic chamber 60 cm above the floor using adhesive tape placed <1 cm from the tip of the tail. The resulting behavior was recorded for 6 min using a video camera, and the immobility time was measured. In this test, the immobility time was defined as the interval when the mice stopped struggling for ≥1 s. Data acquisition and analysis were performed using the ANY‐MAZE software.

### 2.13 Porsolt forced swim test

The Porsolt forced swim test (PFST) was also used to examine depressive‐like behavior (Ueno et al., 2022). The apparatus consisted of four Plexiglas cylinders (20 cm height × 10 cm diameter). The cylinders were filled with water (23°C) to a depth of 7.5 cm, based on previous studies (Ueno et al., 2022). The mice were placed in the cylinders for 6 min, and their behavior was recorded. Similar to the tail‐suspension test, immobility time was evaluated using the ANY‐MAZE software.

### 2.14 Statistical analyses

Data were analyzed using one‐way analysis of variance (ANOVA) followed by Tukey's test, two‐way repeated measures ANOVA followed by Fisher's least significant difference test, Student's *t*‐test, or paired *t*‐test. Differences were regarded as statistically significant at *p* < .05. Data are presented as box plots.

## 3 RESULTS

### 3.1 General characterization of ApoEshl mice

**FIGURE 2:**
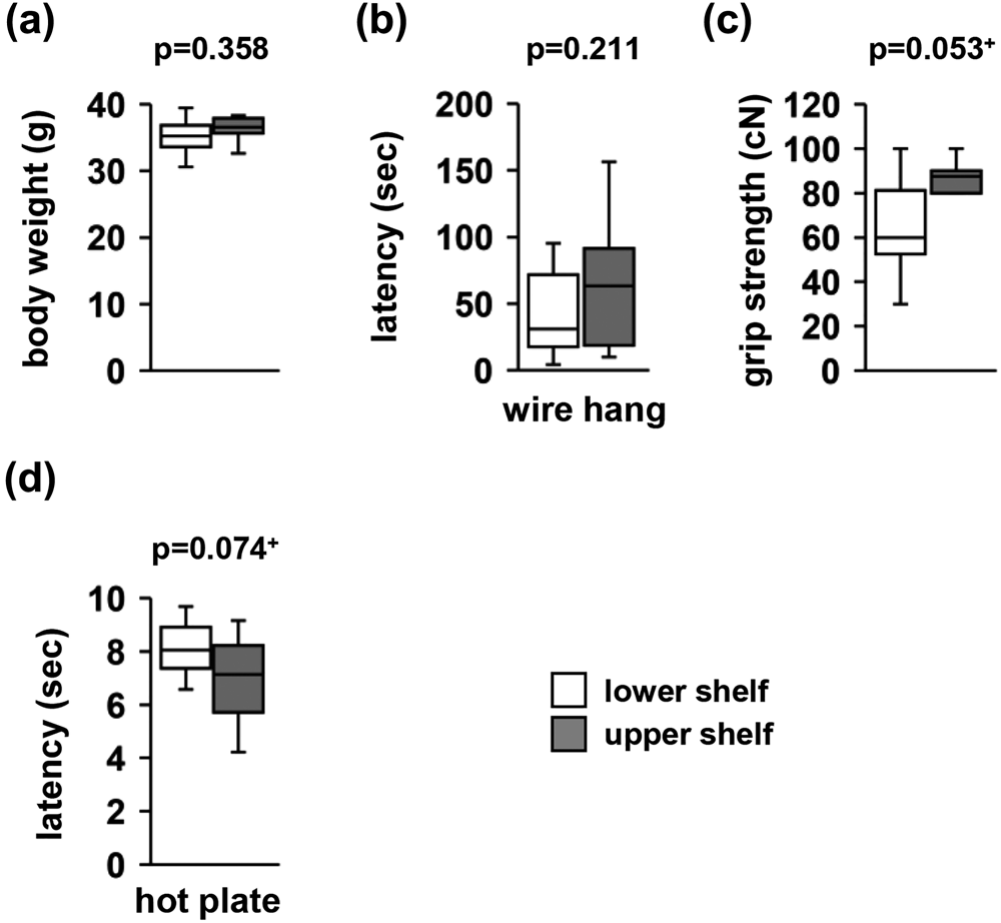
Effects of different heights on physical characteristics: (a) body weight, (b) latency to fall in the wire hang test, (c) grip strength, (d) hot plate test. Data are presented as box plots (a–d). Statistical significance is represented by asterisks: *p < .05, ^+^ *p* < .1. The *p* values were calculated using a one‐way analysis of variance (ANOVA) (a–d). (a–d) Upper shelf group: *n* = 10; lower shelf group: *n* = 10.

There were no significant between‐group differences in body weight (Figure 2a, *F*_1,18_ = .891, *p* = .358), the latency to fall in the wire hang test (Figure 2b, *F*_1,18_ = 1.678, *p* = .211), grip strength (Figure 2c, *F*_1,18_ = 4.273, *p* = .053^+^), or the pain threshold in the hot plate test (Figure 2d, *F*_1,18_ = 3.611, *p* = .074^+^).

### 3.2 Elevated plus maze test

**FIGURE 3:**
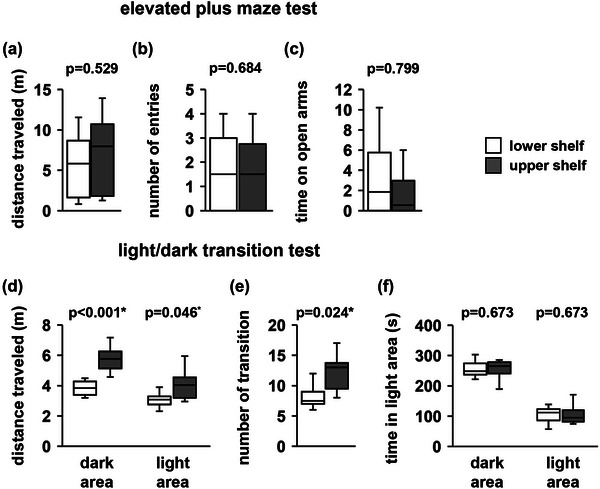
Elevated plus maze test and the light/dark transition test performance. Elevated plus maze test: total distance traveled (a), number of total entries into open arms (b), and time spent in the open arms (c). Light/dark transition test: distance traveled in the dark and light areas (d), number of light/dark transitions (e), and time spent in the dark and light areas (f). Data are presented as box plots (a–f). Statistical significance is represented by asterisks: *p < .05, ^+^ *p* < .1. *p* Values were calculated using a one‐way (a–c, e) or two‐way analysis of variance (ANOVA) (d and f). (a–f) Upper shelf group: *n* = 10; lower shelf group: *n* = 10.

Anxiety‐like behavior was evaluated using the elevated plus maze test. There were no significant between‐group differences in the distance traveled (Figure 3a, *F*_1,18_ = .411, *p* = .529), total number of entries into open arms (Figure 3b, *F*_1,18_ = .171, *p* = .684), or total time spent in the open arms (Figure 3c, *F*_1,18_ = .067, *p* = .799).

### 3.3 Light/dark transition test

Upper shelf mice showed decreased anxiety‐like behavior as reflected by a significantly higher total distance traveled in the light area (Figure 3d, dark area: *p* < .001*, light area: *p* = .046*) and a significantly higher total number of transitions compared to lower shelf mice (Figure 3e, *F*_1,18_ = 6.056, *p* = .024*). There were no between‐group differences in the total time spent in either the dark or light areas (Figure 3f, dark area: *p* = .673, light area: *p* = .673).

### 3.4 Open field test

**FIGURE 4:**
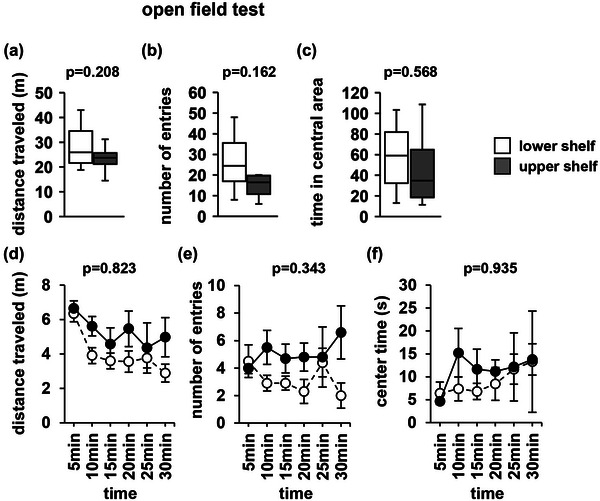
Open field test performance. Total distance traveled (a), number of entries into the central area (b), and total time spent in the central area (c). Graphs showing the distance traveled (d), number of entries into the central area (e), and time spent in the central area (f) in each of the 5 min test periods. Data are presented as box plots (a–c) or as mean ± standard error (d–f). Statistical significance is represented by asterisks: *p < .05, ^+^ *p* < .1. *p* Values were calculated using a one‐way (a–c) or two‐way repeated‐measures analysis of variance (ANOVA) (d–f). (a–f) Upper shelf group: *n* = 10; lower shelf group: *n* = 10.

In the open field test, there were no significant between‐group differences in the total distance traveled (Figure 4a, *F*_1,18_ = 1.702, *p* = .208), number of entries into the central area (Figure 4b, *F*_1,18_ = 2.123, *p* = .162), or time spent in the central area (Figure 4c, *F*_1,18_ = .337, *p* = .568). Similarly, there were no significant between‐group differences in the distance traveled (Figure 4d, *F*_1,108_ = .435, *p* = .823), number of entries into the central area (Figure 4e, *F*_1,108_ = 1.141, *p* = .343), or time spent in the central area (Figure 4f, *F*_1,108_ = .258, *p* = .935) in any 5‐min period.

### 3.5 *Y*‐maze test

**FIGURE 5:**
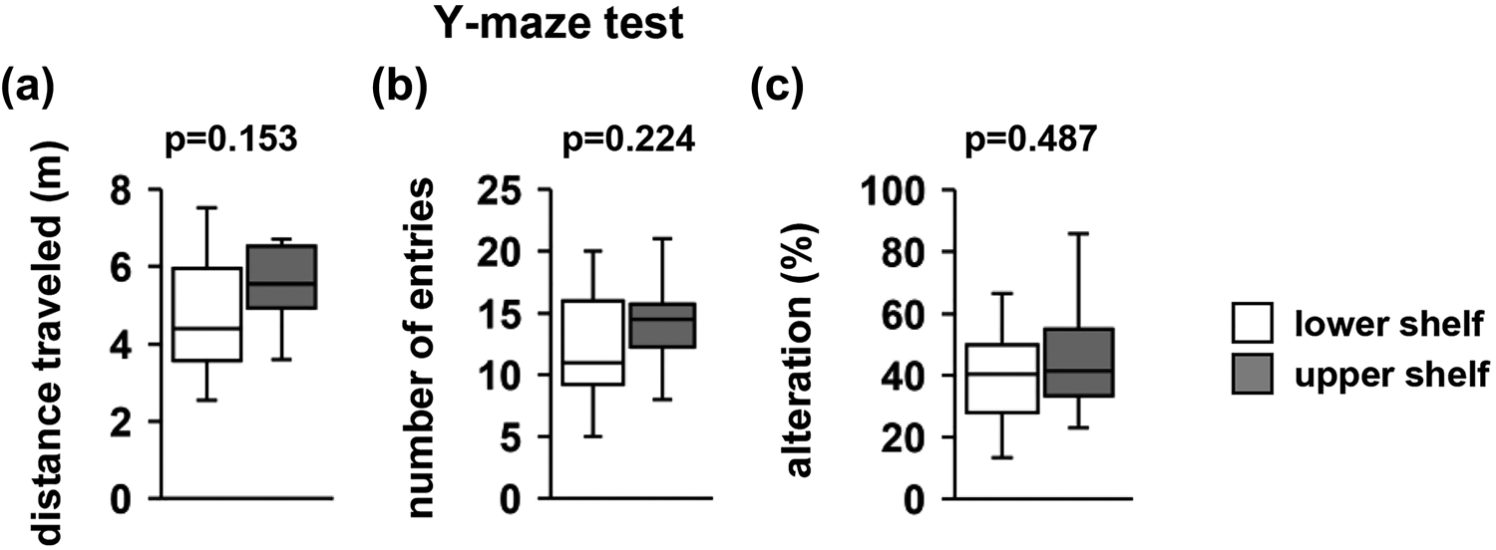
*Y*‐maze test performance. Total distance traveled (a), total number of arm entries (b), and percentage of alternations (c). Data are presented as box plots (a–c). Statistical significance is represented by asterisks: *p < .05, ^+^ *p* < .1. *p* Values were calculated using a one‐way analysis of variance (ANOVA) (a–c). (a–c) Upper shelf group: *n* = 10; lower shelf group: *n* = 10.

In the *Y*‐maze test, there were no significant between‐group differences in the total distance traveled (Figure 5a, *F*_1,18_ = 2.227, *p* = .153), number of arm entries (Figure 5b, *F*_1,18_ = 1.587, *p* = .224), or percentage of alternations in the total number of entries (Figure 5c, *F*_1,18_ = .503, *p* = .487).

### 3.6 Tail‐suspension and Porsolt forced swim test

**FIGURE 6:**
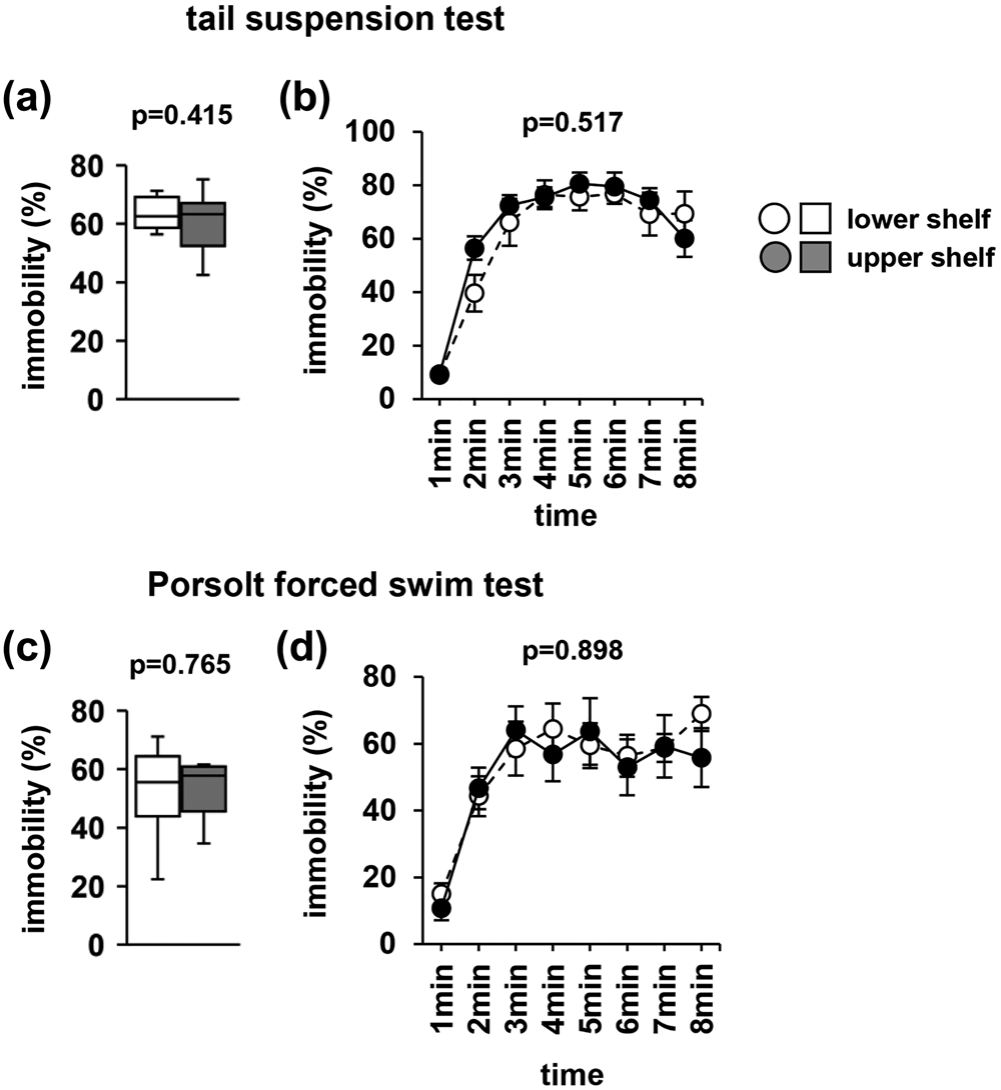
Tail‐suspension test and Porsolt forced swim test performance. Tail‐suspension test: the proportion of total time spent immobile (a) and the proportion of time spent immobile in each 1‐min period (b). Porsolt forced swim test: the proportion of total time spent immobile (c) and the proportion of time spent immobile in each 1‐min period (d). Data are presented as box plots (a, c) or as mean ± standard error (b, d). Statistical significance is represented by asterisks: *p < .05, ^+^ *p* < .1. *p* Values were calculated using a one‐way (a, c) or two‐way repeated‐measures analysis of variance (ANOVA) (b and d). (a–d) Upper shelf group: *n* = 10; lower shelf group: *n* = 10.

In the tail‐suspension test, there were no significant between‐group differences in the total immobility time (Figure 6a, *F*_1,18_ = .695, *p* = .415) or immobility time percentage in any 1‐min period (Figure 6b, *F*_1,144_ = .889, *p* = .517). Similarly, in the PFST, there were no significant between‐group differences in the total immobility time (Figure 6c, *F*_1,18_ = .092, *p* = .765) or immobility time percentage in any 1‐min period (Figure 6d, *F*_1,144_ = .405, *p* = .898).

## 4 DISCUSSION

In this study, we investigated behavioral changes in mice raised at different heights for 3 weeks. Mice housed on high shelves showed reduced anxiety‐like behavior in the light/dark transition test compared to mice housed on low shelves. There were no significant differences between the two groups in terms of activity, exploratory behavior, muscle strength, or depression‐like behavior. These results indicate that mice housed in cages of different heights behave differently in this experimental environment.

The reproducibility of experimental studies in biomedical research has been a topic of intense debate over the last decade (Baker, 2016; Fitzpatrick et al., 2018). In fact, the nonreproducible prevalence has been estimated to range from 50% to 90% (Begley & Ellis, 2012; Prinz et al., 2011). Animal models are unique sources of in vivo data in many areas of biomedical research, and mice have been the most widely used laboratory animals for the study of disease, behavior, and pharmacology over the last century (Taylor et al., 2008). To successfully transfer the results from mice to human experiments, it is necessary to standardize and properly report the appropriate treatment, handling, and experimental methods of laboratory mice.

In general, depression‐like behavior, aggression, activity, and anxiety‐like behavior in mice are measured through a series of behavioral experiments to analyze the effects of drugs and other stimuli (Wahlste, 2010). Behavioral test batteries have been used to assess a variety of behavioral traits, including motor activity, sensory and motor function, anxiety‐like behavior, learning, and memory in inbred and mutant mouse strains (Crawley, 2008; Crawley & Paylor, 1997). Therefore, although behavioral experiments are generally designed to minimize the potential effects of various confounding factors, reproducibility remains an issue.

In the present study, mice housed on shelves of different heights did not show significant differences in body weight. In a previous study, mice housed in high rows of racks have shown evidence of increased stress physiology, immune function, and stress (Theil et al., 2020). However, the details of weight and behavioral changes have not yet been clarified. Stress is well known to alter body weight and food intake in animal models. Daily restraint stress causes decreased food intake and weight loss in ICR male mice (Jeong et al., 2013). It has also been reported that chronic restraint stress reduces body weight in other mouse strains (Shoji & Miyakawa, 2020; Woo et al., 2018). Potentially, our results did not reveal any weight differences because the mice were not stressed.

Wire hanging is an optimal method for measuring muscle coordination and endurance in mice (Rogers et al., 1997). No significant differences were found between the two groups in the wire hanging test that suggests that the height of the rearing shelf does not affect the muscle strength of mice.

Analgesia was measured using the hot plate test, a classic assay that reflects different modalities of thermal nociception (Tjolsen & Hole, 1997). In our experiment, we found no significant differences between the two groups in the hot plate test, which suggests that cage elevation has no effect on pain sensitivity.

The open field test is a behavioral experiment that measures anxiety‐like behavior in open spaces (Jin et al., 2018; Seibenhener & Wooten, 2015). The light/dark transition test is a behavioral experiment used to measure anxiety in bright places (Bourin & Hascoët, 2003; Takao & Miyakawa, 2006). The elevated plus maze test is a behavioral experiment to measure anxiety in high places (Holmes et al., 2000; Komada et al., 2008; Rodgers & Dalvi, 1997). There are various types of anxiety‐like behaviors, such as anxiety about heights, bright places, and large objects. Mice housed on shelves of different heights did not show differences in anxiety‐like behavior in the open field or elevated plus maze tests. We expected that home‐cages on high shelves would attenuate anxiety‐like behaviors at high altitudes; however, no differences were observed. In the light/dark transition test, the mice housed on high shelves showed reduced anxiety‐like behavior that suggests that mice raised on high shelves have reduced anxiety regarding bright light.

In the current experiment, the light intensity was higher on the high shelf than on the low shelf. Previous studies have also shown that animals in high rows may be exposed to bright light for long periods of time (Theil et al., 2020). Mice are highly sensitive to light and have been entrained to light levels around 0.01 photopic lux (Butler & Silver, 2011; Ebihara & Tsuji, 1980). There is some variability among mouse strains, with the C57 mouse strain being more susceptible than the C3H (Foster & Helfrich‐Förster, 2001). The significant variability of light levels between mouse cage racks has been shown to cause potential experimental variability (Steel et al., 2022). Animals housed in blue and amber cages have perturbed rhythms in endocrine metabolism and physiological measures such as plasma corticosterone levels compared to those housed in clear cages (Dauchy et al., 2013). Light has powerful effects on mouse physiology and behavior, including activity levels (Hughes et al., 2015), sleep and wakefulness (Pilorz et al., 2016), body temperature (McGuire et al., 1973), melatonin production (Brainard et al., 1984), and corticosterone secretion (Ishida et al., 2005). Therefore, differences in home‐cage light levels across experimental cages are a potential source of biological variability, including variability in exploratory activity, anxiety, and depression‐like behaviors. In this study, shelves of different heights caused differences in light levels in the home‐cages which indicate that the difference in light levels changed the anxiety‐like behavior of the mice toward bright places. This study found no significant differences between groups in the open field test or elevated plus maze test. This may be due to the illuminance during the behavioral test and the order of the behavioral tests. Experiments are needed to examine these behavioral tests under different illuminances and sequences. In addition, it has been reported that C57BL/6N strain mice do not exhibit increased anxiety‐like behavior with increased light exposure compared with C57BL/6J mice (Capri et al., 2019). Furthermore, another possible way to discern the shelf and light effects would be to add the C3H mouse strain which is visually blind but has melatonin to see if visual perception of light affects the behavior in the light‐dark box test. Further studies using the C3H mouse strain are needed.

Light levels experienced by the animals in the rack depend on the position and material of the cage. Both rows and columns of cages can affect light intensity, depending on how the racks are positioned relative to room lighting (Clough, 1982). Rats and mice generally prefer cages with low light intensities (Blom et al., 1996). A room light level of approximately 325 lx and a height of approximately 1 m above the floor are standard guidelines (National Research Council, 2011). Minimizing light intensity variability between cages on a rack is highly recommended to maintain the illumination intensity at an appropriate level and minimize experimental variability (Castelhano‐Carlos & Baumans, 2009). To control for this potential source of variability, investigators should consider cage position in their experimental design to account for differences in the light intensity. The current study shows that researchers need to consider not only room lighting, but also the light levels experienced by animals in their cages when designing experiments. The use of a battery of standardized behavioral tests is necessary to ensure a more accurate interpretation of behavioral phenotypes. If there is a way to control lighting levels on the different shelves (LED lights hanging above each level, lighting below), there would be no need to consider the effects of cage position.

Adult male mice were used in this study. The experimental results may change if developing or female mice, or different mouse strains are used. Further research is needed to clarify the effects of shelf height and light levels in rearing cages on mouse behavior.

## 5 CONCLUSIONS

In this experimental environment, mice housed on shelves of different heights exhibited different behaviors. In particular, those housed on high shelves experienced high light intensity and, thus, showed attenuated anxiety‐like behavior toward bright places. Researchers should carefully control and report the cage elevation and light intensity experienced by laboratory mice.

## AUTHOR CONTRIBUTIONS

All authors had full access to all study data and take full responsibility for the integrity of the data and the accuracy of the analysis. *Study concept and design*: Hiroshi Ueno, Motoi Okamoto, and Takeshi Ishihara. *Acquisition of data*: Hiroshi Ueno and Yu Takahashi. *Analysis and interpretation of data*: Hiroshi Ueno and Yu Takahashi. *Drafting of the manuscript*: Hiroshi Ueno and Motoi Okamoto. *Critical revision of the manuscript for important intellectual content*: Shinji Murakami, Kenta Wani, Yu Takahashi, Yosuke Matsumoto, and Takeshi Ishihara. *Statistical analysis*: Hiroshi Ueno and Yu Takahashi. *Study supervision*: Motoi Okamoto and Takeshi Ishihara.

## CONFLICT OF INTEREST STATEMENT

The authors declare no conflicts of interest.

## FUNDING INFORMATION

This research did not receive any specific grants from funding agencies in the public commercial or not‐for‐profit sectors.

### PEER REVIEW

The peer review history for this article is available at https://publons.com/publon/10.1002/brb3.3269.

## Data Availability

The datasets generated and analyzed during the current study are not publicly available but they are available from the corresponding author upon reasonable request.
